# Prevalence of ineffective breastfeeding technique and associated factors among lactating mothers attending public health facilities of South Ari district, Southern Ethiopia

**DOI:** 10.1371/journal.pone.0228863

**Published:** 2020-02-11

**Authors:** Gizachew Yilak, Woiynshet Gebretsadik, Hiwot Tadesse, Megbaru Debalkie, Agegnehu Bante

**Affiliations:** 1 Department of Nursing, College of Medicine and Health Sciences, Mizan Tepi University, Mizan, Ethiopia; 2 Department of Nursing, College of Medicine and Health Sciences, Arba Minch University, Arba Minch, Ethiopia; Emory University School of Public Health, UNITED STATES

## Abstract

**Introduction:**

Improper positioning, attachment, and suckling are constructs for ineffective breastfeeding technique (IBT). IBT results in inadequate intake of breast milk, which leads to poor weight gain, stunting, and declines immunity. Besides, IBT increases the risk of postpartum breast problems. Despite its impact on maternal and child health, breastfeeding technique is not well studied in Ethiopia. Hence, the purpose of this study was to assess the prevalence of IBT and associated factors among lactating mothers attending public health facilities of South Ari district, Southern Ethiopia.

**Materials and methods:**

An institution-based cross-sectional study was conducted among 415 lactating mothers attending public health facilities of South Ari district from March 1-29, 2019. A structured observational checklist and interviewer-administered questionnaires were used. Bivariable and multivariable analyses were carried out using binary logistic regression to assess the association between explanatory variables and IBT. Statistical significance was declared at p-value < 0.05.

**Results:**

Overall, the prevalence of IBT was 63.5% [95% confidence interval (CI); 59.0%, 68.0%]. Having no formal education [adjusted odds ratio (AOR): 5.0, 95% CI: 2.3, 10.5], delivering at home [AOR: 4.5; 95% CI; 1.6, 13.1], having breast problems [AOR: 2.5, 95% CI: 1.1, 5.7], being primiparous [AOR: 1.8, 95% CI: 1.0, 3.2], not receiving counseling during pregnancy and postnatal period [AOR: 2.3, 95% CI: 1.4, 3.9 and AOR: 2.5, 95% CI: 1.3, 5.1 respectively] were significantly associated with IBT.

**Conclusion:**

IBT was very high in the study area. Thus, empowering women, increasing institutional delivery, and providing continuous counseling about breastfeeding throughout the maternal continuum of care is invaluable to improve breastfeeding techniques.

## Introduction

Breast milk provides the ideal nutrition for infants; nearly a perfect mix of vitamin, protein, fat, and antibodies [1–3]. Breastfeeding is imperative for the mother to facilitate involution [4], reduce the risk of breast cancer [5], epithelial ovarian cancer [6], osteoporosis [7], and coronary artery disease [8]. Ensuring universal breastfeeding can avert 823,000 under-five children deaths and 20,000 breast cancer deaths every year [9]. However, most mothers do not realize breastfeeding technique as a learned skill that needs practice and patience [10].

Breastfeeding technique is the composite of positioning, attachment, and suckling. Positioning denotes the technique in which the infant is held in relation to the mother's body. Attachment indicates whether the infant has enough areola and breast tissue in the mouth, and suckling denotes to the drawing of milk into the mouth from the nipple [11]. Improper positioning, attachment, and suckling are constructs for ineffective breastfeeding technique (IBT), which results in inadequate intake of breast milk that leads to poor weight gain, stunting, and declines immunity [10, 12]. The 2019 mini-Ethiopian demographic health survey (MEDHS) reported that 37%, 21% and 7% of under-five children are stunted, underweight and wasted respectively [13]. IBT is also the leading cause of breast engorgement, cracked nipple, mastitis, and breast abscess [14, 15].

Although there is a significant reduction in the global child mortality over the past three decades, around 5.3 million under-five children died in 2018; sub-Saharan Africa contributes ~50% of these deaths [16]. Suboptimal breastfeeding practices increase the risk of infant mortality; non-breastfed and partially breastfed infants are 14 and 3 times higher risk of death in the first six months as compared to exclusively breastfed infants [17]. Moreover, poor positioning increases sufferings from diarrheal and respiratory infections [18]. IBT results in a reduction of exclusive breastfeeding (EBF) practice [11]. Even though there are improvements over the past fourteen years, in Ethiopia only 59.0% of infants under 6 months are engaged in EBF [13].

The prevalence of IBT ranges from 30-70% in Denmark, Brazil, Nepal, India, Libya, and Ethiopia [15, 19–23]. Breast problems, low level of maternal education, lack of breastfeeding experience, home delivery, insufficient counseling, operative deliveries, and primiparity are some of the factors that contribute to IBT [11, 15, 21].

Despite effective breastfeeding techniques have a proven short and long term benefit both for the mother and her child, its practice is not satisfactory in developing countries [24]. Studies that have assessed factors associated with IBT have been mainly conducted in middle and high-income countries [15, 19, 20, 22, 23]. Additionally, such studies have been conducted immediately after birth before the mother is stable and comfortable which may ultimately influence breastfeeding techniques. As such, there is a paucity of data on IBT and its associated factors in developing countries. Furthermore, considering the time of observing breastfeeding techniques, especially not immediately after birth, would ensure that breastfeeding techniques are observed while mothers are stable and comfortable. Besides, there is a dearth of evidence concerning IBT in southern Ethiopia. Hence, this study was initiated to determine the prevalence of IBT and associated factors in South Ari district, Southern Ethiopia.

## Materials and methods

### Study area, design and period

An institution-based cross-sectional study was conducted from March 1-29, 2019 at public health facilities of the South Ari district. South Ari district is one of the districts in the south Omo zone and it is located 17 km away from Jinka (i.e. the capital of the zone) and 771 Km away from Addis Ababa, the capital city of Ethiopia. The population of the district is around 254,000; of this, 8,788 were reproductive-age women. The district had one district hospital and eight public health centers that provide delivery, postnatal and immunization services for the catchment area population.

### Study population

All mothers who visited public health facilities of South Ari district for immunization and/or postnatal care services were considered as the study population. Lactating mothers who had under six-month infants were included. Those mothers who were seriously ill and unable to breastfeed their newborn, whose infants were critically ill and neonates with major congenital cleft lip and cleft palate were excluded from the study.

### Sample size determination

The sample size was calculated by EpiInfo-7 StatCalc using a single population proportion formula using the following assumptions: 57.0% (prevalence of IBT from a study conducted in Harar, Ethiopia [21], 95% level of confidence, and 5% margin of error. By adding a none response rate of 10% the final sample size was 415.

### Sampling technique

**Fig 1:**
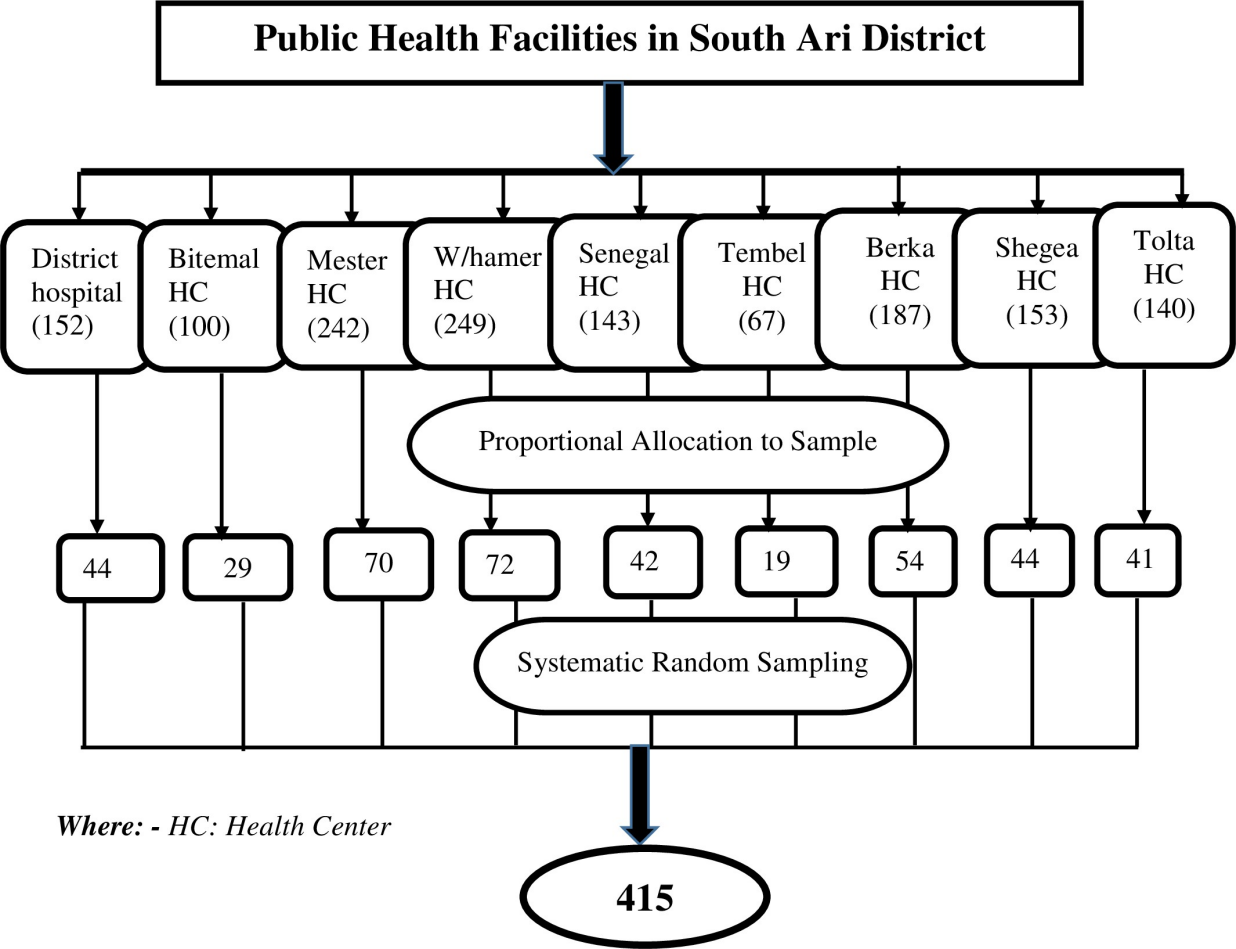
Schematic presentation of the sampling procedure for the study conducted among lactating mothers attending public health facilities of South Ari district, Southern Ethiopia, 2019.

All public health facilities in South Ari district (one district hospitals and eight health centers) were included in this study and the sample size was allocated proportionally to each health facility based on the number of postnatal care (PNC) and immunization service attendants in the last quarter of 2018. Based on the review, the total number of lactating mothers who came for immunization and PNC services were 1433. Then, to get the sampling interval total number of lactating mothers who came for immunization and PNC in the last quarter of 2018 was divided by the number of the required sample size, which is 1433/415=3. Finally, the data were collected by using a systematic random sampling technique with an interval of three (k =3) **(Fig 1)**.

### Data collection tool

A structured observational checklist and interviewer-administered questionnaire were prearranged after reviewing previous literature [15, 21]. The tool comprises socio-demographic, maternal and infant characteristics. The WHO B-R-E-A-S-T-Feed observational checklist was used to assess mother and baby’s position, infant’s mouth attachment, and suckling. According to WHO criteria, four components (i.e. baby body should be straight and slightly extended, baby body close and turned toward the mother, the whole body supported, and baby facing toward the mother’s breast) are used to assess the baby’s position in relation to the mother’s body. Likewise, attachment of the baby to the breast was assessed by four components: more areola is visible above the baby’s upper lip, the baby’s mouth is wide open, the baby’s lower lip is turned outward and the baby’s chin is touching or almost touching the breast. Furthermore, suckling was assessed by three components: slow sucks, deep suckling and sometimes pausing [25, 26].

### Data collection procedure

Eight diploma midwives and three bachelors of science midwives were recruited as data collectors and supervisors, respectively. A 2-days training about the purpose of the study and process of data collection was given to the data collection team. The positions of the mother and baby, infant’s mouth attachment to breast and suckling were observed through hands-on practice. Data collectors observe the breastfeeding process for five minutes and record as per the WHO B-R-E-A-S-T feed observation form. The observation was done by asking the mother to put her infant to the breast. When the infant was fed in the previous hour, the mother was kindly asked to stay away for a few minutes and observation of the breastfeeding technique was done during the next time when the baby was ready to feed. Data collection, supervision was carried out on a daily basis throughout the study period.

### Study variables and measurements

Ineffective breastfeeding technique, obtained from three composite variables (i.e. Positioning, attachment, and suckling), was the outcome variable (**Table 1**). Furthermore, socio-demographic, maternal and infant characteristics were the independent variables for this study. Maternal age, in completed years, was categorized and coded into four groups as ‘< 20 years’= “1”, ‘20-25 years’= “2”, ‘26 -30 years’ = “3” and ‘> 30 years’= “4”. Maternal level of education was categorized and coded into three as ‘no formal education’ = “1”, ‘attend primary education (grade 1-8)’= “2”, and ‘secondary education and above (grade nine and above)’ = “3”. Parity, the total number of live births after the age of viability (28 completed weeks of gestation), was categorized and coded as ‘primipara’ = “1” and ‘multipara’ = “2”. Antenatal care (ANC) was categorized and coded into two as ‘Yes’ = “1” and ‘No’ = “2”.

**Table 1:** Description of variables and measurements for the study in South Ari District, Southern Ethiopia, 2019.

Variables	Descriptions	Measurement/Category
****Dependent/outcome Variable****		
Ineffective breastfeeding (IBT)	IBT was a composite variable of the three constructs (positioning, attachment, and suckling) such that lactating women with at least one of the constructs categorized as “poor” were regarded as having IBT [21].	Those mothers with IBT were categorized as ‘Yes’ and labeled as “1” and mothers with effective breastfeeding techniques were categorized as ‘No’ and labeled as “0”.
****Composite Variables****		
Positioning	The technique in which the infant is held in relation to the mother's body. Good positioning: when at least three out of four criteria for infant positioning was fulfilled; average positioning: when any two of the four criteria were correct and poor positioning when only one or none criterion has been fulfilled [25, 27].	In the beginning, it was categorized as good, average and poor positioning. Then, to create a dummy variable good and average positioning were merged as ‘good’ and labeled as “0” and ‘poor positioning” was coded as it is and labeled as “1”.
Attachment	It indicates whether the infant has enough areola and breast tissue in the mouth. Good attachment: when at least three out of four criterions have been fulfilled. Average attachment: when any two of the four criterions have been fulfilled. Poor attachment: when only one or none out of four criterions have been fulfilled [21, 27].	Like that of positioning, first, it was categorized as a good, average and poor attachment. Then, to create a dummy variable good and average attachment were merged as ‘good attachment’ and labeled as “0” and ‘poor attachment” was coded as it is and labeled as “1”.
Suckling	Drawing of milk into the mouth from the nipple. Effective suckling: at least two out of three criterions have been fulfilled. Ineffective suckling: only one or none from three criterions has been fulfilled [25, 27].	Suckling was coded as ‘effective suckling’, labeled as “0” and ‘ineffective suckling’, labeled as “1”.

Prenatal counseling, counseling rendered to pregnant women about birth preparedness and complication readiness, breastfeeding and other postpartum related complications, was categorized into two as those mothers who received counseling were categorized and coded as ‘Yes’= “1” and those who didn’t receive were categorized and coded as ‘No’ = “2”. Likewise, Postnatal counseling, service designed immediately after delivery to provide emotional support for mothers and their partners about breastfeeding, postpartum related complications, and safe motherhood, was categorized into two; those who received counseling were coded as ‘Yes’= “1” and those who didn’t receive were coded as ‘No’ = “2”. Place of delivery, the place where an infant was born, was categorized and coded into three as ‘hospital’ =”1”, ‘health center’ = “2” and ‘home’ = “3”. Breast problems such as breast engorgement, mastitis, and/or breast abscess experienced by women during the postpartum period. It was categorized into two as those who faced any type of breast problems were categorized and coded as ‘Yes’ = “1” and those who didn’t encounter it was categorized and coded as ‘No’ = “2”. Residence, the place where the respondent lives, categorized and coded into two as urban = “1” and rural = “2”. Occupation, the current employment status and specific career of the participant, was categorized and coded into two as housewives = “1” and other = “2”. Other includes government employees, NGO workers, daily laborers, and self-employees. Birth weight, bodyweight of a baby at its birth, was categorized and coded into three as low birth weight (<2500 gram) = “1”, normal (2500-3999 gram) = “2”, and macrosomia (>4000 gram) = “3”. Mode of delivery, childbirth delivery options that natural unassisted childbirth, vaginal operative deliveries and delivery by cesarean section, was categorized into two as normal = “1”, and cesarean section = “2”.

### Data quality control

Rigorous training about the objectives of the study and the procedure to be followed during data collection was given for the data collectors. Structured and validated checklists were adopted from the WHO B-R-E-A-S-T feed observation form [28] and translated into local languages (i.e. Arigna and Amharic). The tool was pre-tested among 21 mothers from the kako health center. Close supervision was undertaken on a daily basis throughout the study period. Double data entry was done on 5% of the sample by two data clerks and consistency of the entered data were cross-checked by comparing the two separately entered data sets.

### Data management and analysis

The data were visually checked by the investigators and entered to EpiData statistical software version 3.1. Then, the data were exported to SPSS version 25.0 for cleaning and analysis. Descriptive summary measures such as frequency, percentages, mean and standard deviation were used to describe characteristics of the participants. Binary logistic regression was carried out to identify the factors associated with IBT. To control possible confounding factors, variables with a p-value of ≤0.25 in the bivariate analysis were taken to the multivariable analysis. Multicollinearity and model fitness was checked using standard error and Hosmer-Lemeshow test respectively. The adjusted odds ratio (AOR), with 95% confidence intervals (CI), was used to identify the independent variables associated with IBT. All tests were two-sided and statistical significance was declared at P-value < 0.05.

### Ethical consideration

Ethical approval was obtained from Arba Minch University, College of medicine and health sciences, Institutional Ethical Review Board (IRB). Permission was secured from the hospital and health center administrators. Moreover, voluntary informed verbal consent was obtained from the study participants before the initiation of the data collection. Code numbers were used throughout the study to maintain the confidentiality of information gathered from each study participant.

## Results

### Socio-demographic characteristics

A total of 414 participants were involved, making a response rate of 99.8%. The mean (± standard deviation (SD)) age of the participants was 26.5 (±5.2 SD) years. Of the participants, 31.9% were within the age group of 26-30 years, and 66.9% were protestant by religion. The majority of the participants, 86.3% were married, 69.1% were housewives, 63.3% were rural dwellers, and 60.8% didn’t attend formal education **(Table 2).**

**Table 2:** Sociodemographic characteristics of participants attending public health facilities of South Ari district, Southern Ethiopia, 2019 (n= 414).

Variables	Frequency	Percentage (%)
****Age****		
< 20	72	17.4
20–25	124	30.0
26–30	132	31.9
> 30	86	20.7
****Ethnicity****		
Ari	319	77.1
Amhara	52	12.6
Wolayita	31	7.5
Other^a^	12	2.8
****Religion****		
Protestant	277	66.9
Orthodox	96	23.2
Muslim	25	6.0
Other^b^	16	3.9
****Level of education****		
No formal education	252	60.8
Primary school	100	24.2
Secondary school and above	62	15.0
****Occupation****		
Housewife	286	69.1
Government employee	38	9.2
Self-employed	42	10.1
Daily laborer	33	8.0
Other^c^	15	3.6
****Marital Status****		
Married	357	86.3
Single	20	4.8
Divorced	24	5.8
Other^d^	13	3.1
****Residence****		
Rural	304	73.4
Urban	110	26.6
****Family size****		
< 5	300	72.5
≥ 5	114	27.5

^a^Oromo, konso and Gamo Gofa

^b^Catholic, yewuha miskir

^c^NGO, student, farmer

^d^Widowed, Separated

### Obstetric characteristics

Among the participants, 33.1% were primipara, 95.6% had ANC follow-up, and 93.2% were delivered through the natural route. Four-fifths (80.2%) of the participants received counseling about breastfeeding techniques after delivery. The majority (86.7%) of the participants were delivered at term. The birth weight of the newborns was within the normal range for 92.0% of the participants. Nearly half (46.1%) of the infants were male **(Table 3).**

**Table 3:** Obstetric characteristics of participants attending public health facilities of South Ari district, southern Ethiopia, 2019 (n = 414).

Variables		Frequency	Percentage
Parity	Primipara	137	33.1
	Multipara	277	66.9
Ever had stillbirth	Yes	97	23.4
Ever had a neonatal death	Yes	51	12.3
Antenatal care	Yes	396	95.6
Pregnancy status	Planned	323	78.0
	Unplanned	91	22.0
Received counseling during pregnancy	Yes	245	59.2
Place of delivery	Hospital	123	29.7
	Health center	236	57.0
	Home	55	13.3
Mode of delivery	Normal delivery	383	92.5
	Cesarean section	31	7.5
Received postnatal counseling about BFT*	Yes	332	80.2
Gestational age at delivery	Term	359	86.7
	Preterm	34	8.2
	Post-term	21	5.1
Birth weight	Low birth weight	15	3.7
	Normal	381	92.0
	Macrosomia	18	4.3
Age of the infant	< 42 days	164	39.6
	≥ 42 days	250	60.4
Sex of the Infant	Male	191	46.1
	Female	223	53.9
Breast problem	Yes	59	14.3
Giving pre-lacteal feeding	Yes	26	6.3
Initiate complementary feeding	Yes	137	33.1

*Breastfeeding techniques

### Status of ineffective breastfeeding technique

**Fig 2:**
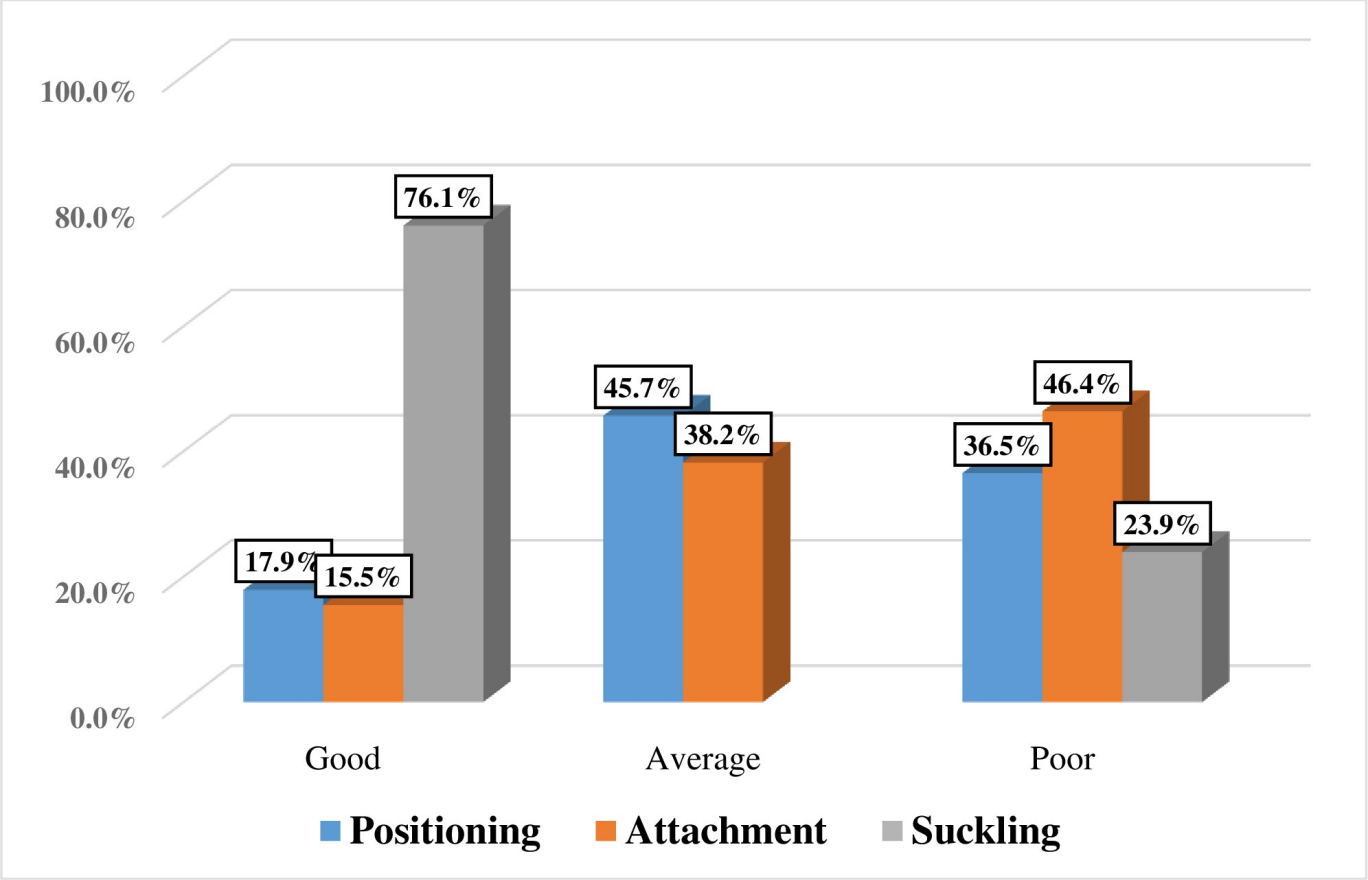
Status of breastfeeding technique constructs in public health facilities of South Ari district, Southern Ethiopia, 2019 (n=414).

**Fig 3:**
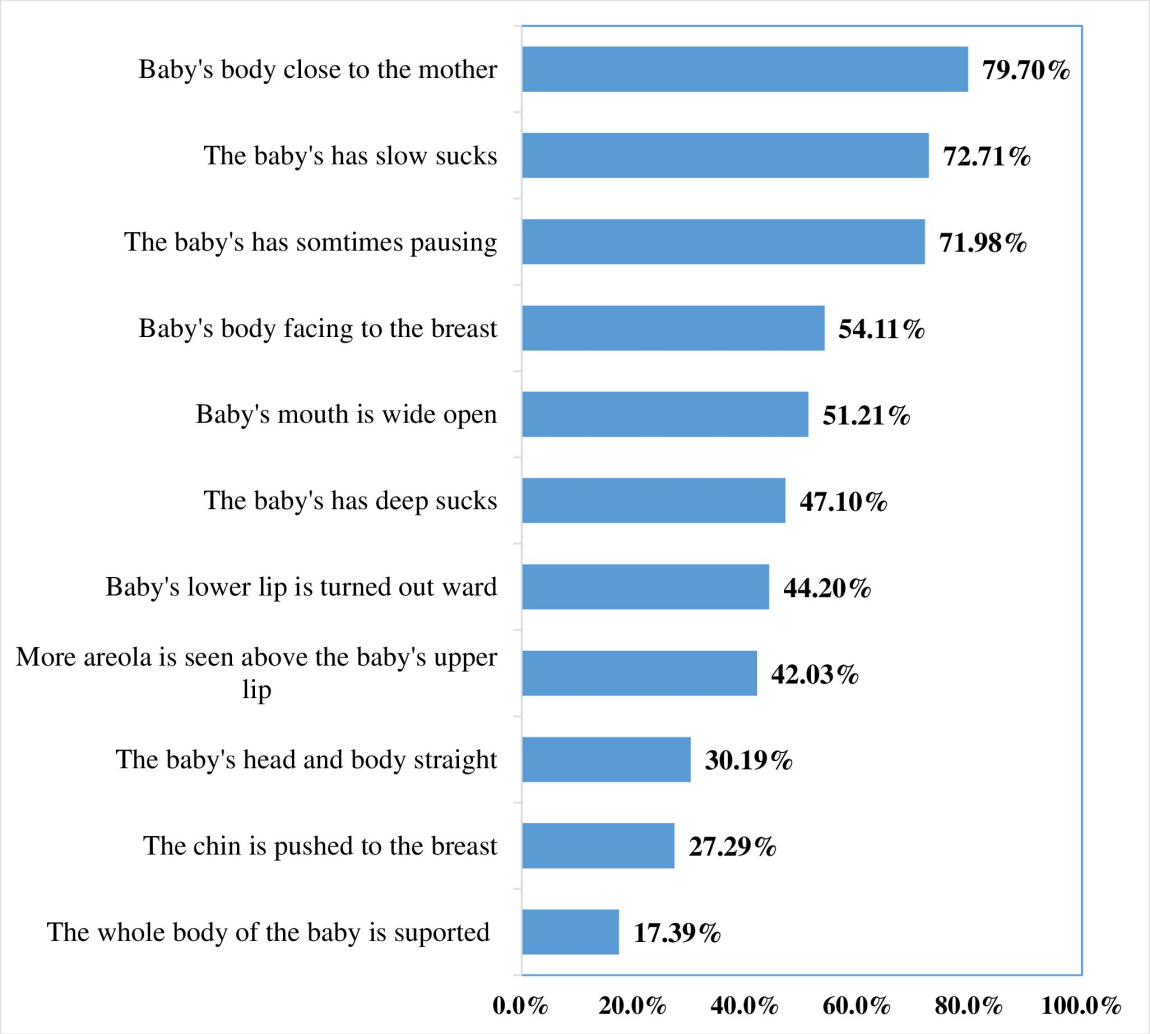
Effective breastfeeding practice for each item in public health facilities of South Ari district, Southern Ethiopia, 2019 (n=414).

Overall, the prevalence of IBT was 63.5% (95%, CI: 59.0%, 68.0%). Poor positioning was observed among 36.4% of women **(Fig 2)**. Only 17.4% of the participants supported the whole body of their baby and the baby’s head and body were straight for 30.2% of the newborns **(Fig 3).**

### Factors associated with ineffective breastfeeding technique

The odds of IBT was 5 times (AOR=5.0; 95% CI: 2.3, 10.5) higher among mothers who did not attend formal education as compared to those who had secondary and above education. IBT was 4.5 times (AOR=4.5; 95% CI; 1.6, 13.1) higher among mothers who delivered at home as compared to mothers who delivered in the hospital. Likewise, as compared to multiparous, the odds of IBT was almost two times (AOR=1.8; 95% CI: 1.0, 3.2) higher among primiparous women. Mothers who had breast problems were 2.5 times (AOR=2.5; 95% CI: 1.1, 5.7) more likely to exhibit IBT as compared to their counterparts. Moreover, the odds of IBT was 2.3 times (AOR=2.3; 95% CI: 1.4, 3.9) and 2.5 times (AOR=2.5; 95% CI: 1.3, 5.1) higher among participants who did not received counseling about breastfeeding techniques during pregnancy and at the postnatal period respectively as compared to their counterparts **(Table 4).**

**Table 4:** Factors associated with ineffective breastfeeding technique in public health facilities of South Ari district, Southern Ethiopia, 2019 (n = 414).

Variables	^a^IBT		^b^COR (95% CI)	P-value	^c^AOR (95% CI)
	Yes (N, %)	No (N, %)			
****Age category in completed years****					
<20	53 (73.6)	19 (26.4)	1.6 (0.8, 3.1)	0.195	1.1 (0.4, 2.7)
20-25	77 (62.0)	47 (38)	0.9 (0.5, 1.6)	0.784	0.9 (0.4, 1.8)
26 -30	78 (59)	54 (41)	0.8 (0.5, 1.4)	0.472	0.8 (0.4, 1.6)
>30 years	55 (64)	31 (36)	1.0		1.0
****Educational status****					
No formal education	178 (70.6)	74 (29.4)	4.7 (2.6, 8.4)	0.0001	5.0 (2.3, 10.5)*
Primary school	64 (64)	36 (36)	3.4 (1.8, 6.7)	0.0001	3.3 (1.4, 7.6)*
Secondary & above	21 (33.8)	41 (66.2)	1.0		1.0
****Parity****					
Primipara	96 (70.1)	41 (29)	1.5 (0.99, 2.4)	0.052	1.8 (1.0, 3.2)*
Multipara	167 (60.3)	110 (39.7)	1.0		1.0
****Antenatal care visit****					
Yes	248 (62.6)	148 (37.4)	1.0	0.088	1.0
No	15 (83.3)	3 (16.7)	2.9 (0.85, 10.4)		2.3 (0.5, 10.6)
****Prenatal counseling about breastfeeding techniques****						
Yes	134 (54.7)	111 (45.3)	1.0	0.0001	1.0
No	129 (76.3)	40 (23.7)	2.7(1.7, 4.1)		2.3 (1.4, 3.9)*
****Place of delivery****					
Hospital	66 (53.6)	57 (46.4)	1.0		1.0
Health center	147 (62.3)	89 (37.7)	1.76 (1.14, 2.7)	0.115	1.01 (0.6, 1.7)
Home	50 (90.9)	5 (9.1)	12.4 (2.8, 54.6)	0.0001	4.5 (1.6, 13.1)*
****Postnatal care counseling about breastfeeding****						
Yes	195 (58.7)	137 (41.3)	1.0		1.0
No	68 (82.9)	14 (17.1)	3.4 (1.8, 6.3)	0.0001	2.5 (1.3, 5.1)*
****Breast problem****					
No	213 (60)	142 (40)	1.0		1.0
Yes	50 (84.7)	9 (15.3)	3.7(1.8, 7.8)	0.001	2.5 (1.1, 5.7)*
****Residence****					
Rural	203 (66.8)	101 (33.2)	1.7 (1.1, 2.6)	0.023	1.7 (0.9, 2.9)
Urban	60 (54.5)	50 (45.5)	1.0		1.0
****Occupation****					
House wife	194 (67.8)	92 (32.2)	1.8 (1.2, 2.8)	0.007	1.3 (0.7, 2.3)
Other^d^	69 (54)	59 (46)	1.0		1.0
****Birth weight****					
Low birth weight	11 (73.3)	4 (26.7)	5.5 (1.2, 24.8)	0.027	2.5 (0.5, 13.2)
Normal	246 (64.6)	135 (35.4)	3.6 (1.3, 9.9)	0.11	2.5 (0.9, 7.3)
Macrosomia	6 (33.3)	12(66.7)	1.0		1.0
****Mode of delivery****					
Normal	247 (64.5)	136 (45.5)	1.7(0.8, 3.6)	0.156	1.3 (0.5, 3.0)
Cesarean delivery	16 (51.6)	15 (48.4)	1.0		1.0

* Significant at p < 0.05

^a^Ineffective breastfeeding

^b^crude odds ratio

^c^adjusted odds ratio

^d^government employee, daily laborer, NGO worker, self-employee

## Discussion

This study revealed that 63.5% of lactating women exhibit IBT. Having no formal education, delivering at home, having breast problems, being primiparous, not receiving counseling during pregnancy and in the postnatal period were significantly associated with IBT.

The prevalence of IBT in this study is higher than the studies conducted in the rural population of India (49%) [22], Cheluvamba hospital, India (57%) [29], Libya (52%) [15] and Harar, Ethiopia (57%) [21]. On the contrary, it is lower than a study conducted in West Bengal/Kolkata hospital India (69.7%) [19]. This discrepancy might be due to the difference in the quality of health services, counseling, and demonstration about breastfeeding techniques during pregnancy and the postpartum period. In addition, it might be due to socio-cultural, study settings and period variation.

The odds of IBT was five times higher among women having no formal education as compared to those who attend secondary and above education. This finding is in line with the studies conducted in Indian East Delhi, West Bengal Kolkata hospital, Saudi Heraa general hospital, Bhaktapur district of Nepal, Sri Lanka and Harare Ethiopia [10, 19, 21, 29–31]. This might be probably due to the fact that uneducated women need much more time to adhere and implement effective breastfeeding techniques. In addition, unschooled mothers may face some difficulties to acquire and observe health information about appropriate breastfeeding practice.

In this study, the likelihood of having IBT was almost two times higher among the primiparous as compared to multiparous mothers. This is in line with the studies conducted in India, Denmark, Cheluvamba Hospital of India, Libya, Harar Ethiopia and Areka town Ethiopia [10, 15, 21, 27, 29]. This might be due to a shortage of information, skill, and experience about breastfeeding techniques. Moreover, multiparous women are more likely to have gained child-rearing experience (including feeding techniques) from their previous pregnancies.

The odds of IBT was three times higher among participants with breast problems as compared to their complements. This is in line with studies conducted in Libya, and Ethiopia [15, 21]. This might be due to the fact that mothers with breast problems may have severe pain that hinders them to apply breastfeeding techniques. In addition, it is difficult to correctly attach the infant’s mouth with engorged and cracked nipples due to distension and edema of the nipple.

Those mothers who did not receive counseling about breastfeeding techniques after delivery were almost three times more likely to exhibit IBT as compared to those who received adequate information. This is consistent with the studies conducted in rural areas of rural areas of Nagpur district, India and Harar Ethiopia [20, 21]. Likewise, the odds of IBT was two times higher among participants who didn’t receive counseling about breastfeeding techniques during pregnancy as compared to their counterparts. This finding is consistent with the studies conducted in Libya and Coastal Karnataka [15, 32]. This might be due to the fact that adequate counseling about breastfeeding during pregnancy and the postpartum period are imperative to achieving effective breastfeeding techniques. Moreover, psychological support given for lactating mothers has a significant effect on breastfeeding technique enhancement [29].

The odds of IBT was five times higher among participants who delivered at home as compared to those who delivered in the hospital. This is in line with the studies conducted in the Bhaktapur district of Nepal and Harar, Ethiopia. [21, 31]. The possible reason is that women who delivered in the hospital are likely to receive continuous psychological and hands-on support to realize effective breastfeeding techniques.

Generally, this study reported the magnitude of IBT using a standardized observational checklist and interviewer-administered questionnaires. Though it is very minimal, observer bias and hawthorn effect might be introduced. To minimize observer bias and hawthorn effect, the data collectors were well trained and qualified, and each study participant was observed in a private setting respectively. Due to the cross-sectional nature of the study, the design could not allow causality to be inferred. Since the study was restricted to public health facilities, it was difficult to generalize to all lactating mothers living in the district. Thus, to minimize the effect of the aforementioned limitations scholars with similar interest are recommended to conduct a community-based study that addresses cultural practices that hinders effective breastfeeding techniques. Overall, the findings from this study are fundamental for policy-makers to design appropriate intervention strategies to improve breastfeeding techniques.

## Conclusions

In the study area, the proportion of IBT was very high. Having no formal education, delivering at home, having breast problems, being primiparous, not receiving counseling during pregnancy and in the postnatal period were significantly associated with IBT. Hence, much work is needed to improve breastfeeding techniques among lactating. Empowering women, increasing institutional delivery, and providing continuous support about appropriate breastfeeding techniques throughout the maternal continuum care is mandatory to come up with a significant reduction in IBT.

## Supporting information

S1 File Data collection tool. (DOCX)

S2 File SPSS cleaned data. (SAV)
