# Supplementary material for: Prevalence of ineffective breastfeeding technique and associated factors among lactating mothers attending public health facilities of South Ari district, Southern Ethiopia
Source: PLoS One. 2020 Feb 11;15(2):e0228863. doi: 10.1371/journal.pone.0228863 (PMC7012449; doi:10.1371/journal.pone.0228863)
Supplement: S1 File — (DOCX) [file pone.0228863.s001.docx]

## English Version Questionnaire

Date of interview (date/month/year): ----------------------------------

Name of the health Institution: --------------------------------------------

Code number of the questionnaire: ------------------------------------

Interviewer’s name & signature: ------------------------------

Supervisor’s name & signature: ------------------------------

**Part-I Socio Demographic Characteristics**

| **S/ no** | | **Questions** | **response and code** | | **Skip** |
| --- | --- | --- | --- | --- | --- |
| **Part I: Socio-demographic Characteristics** | | | | | |
| 101 | | How old are you? ( in completed years) | ______________ | |  |
| 102 | | What is your ethnicity? | 1. 1 Ari 2. 2 Amhara 3. 3 Wolayita 4. 4 Other (specify)---- | |  |
| 103 | | What is your religion? | 1. Protestant  2. Orthodox  3. Muslim  4. Catholic  5. Other (specify)---- | |  |
| 104 | | What is your marital status? | 1. Married  2. Single  3. Divorced  4. Widowed/widower  5. Other(specify) | |  |
| 105 | | What is your occupation? | 1. House wife  2. Government employee  3. NGO  4. Self-employee  5. Daily laborer  6. Other (specify) | |  |
| 106 | | What is your level of education? | 1. Illiterate  2. Read and write  3. Elementary (1^st^ cycle)  4. Elementary (2^nd^ cycle)  5. Secondary Preparatory  6. College/ University | |  |
| 107 | | What is your Residence? | 1 Rural  2 Urban | |  |
| 108 | | Number of families members | ______________ | |  |
| **Part II: Obstetrics and infant characteristics** | | | | | |
| 201 | Number of pregnancy? | | |  |  |
| 202 | Number of live birth after viability? | | |  |  |
| 203 | Do you ever had stillbirth? | | | 1. Yes 2. No |  |
| 204 | Do you ever had neonatal death? | | | 1. Yes 2. No |  |
| 205 | Do you have ANC follow up for last pregnancy | | | 1. Yes 2. No | If no skip to q no 207 |
| 206 | How many times you went to the health  Facility for ANC? | | | ______________ |  |
| 207 | Did pregnancy was planned and supported | | | 1. Yes 2. no |  |
| 208 | Have you get counseling about breast feeding techniques during last pregnancy | | | 1. Yes 2. No |  |
| 209 | Place of delivery of the last pregnancy | | | 1 Hospital  2 Health center  3 Home |  |
| 210 | What was the mode of delivery for the last pregnancy? | | | 1 SVD  2 C/S  3 Assisted delivery |  |
| 211 | If you delivered at health facility have you given immediate postnatal counseling about breast feeding techniques | | | 1 Yes  2 No |  |
| 212 | Have you given pre lacteal feeds | | | 1 Yes  2 No |  |
| 213 | Have you given Supplementary/ complementary feeding? | | | 1 Yes  2 No |  |
| 214 | Did you used pacifier | | | 1 Yes  2 No |  |
| 215 | Presence any breast problem? | | | 1 Yes  2 No | If no skip to q 217 |
| 216 | If yes Specify which | | | 1 crackle nipple  2 Mastitis  3 Engorgement  4 inverted nipple  5 other |  |
| 217 | Gestational age of the infant during birth | | | 1 Term  2 Preterm  3 Post term |  |
| 218 | Sex of the infant | | | 1 Male  2 Female |  |
| 219 | Birth weight | | | ______________ |  |
| 220 | Age of the infant | | | ______________ |  |
| 221 | Birth Weight of the baby (in gm.) | | | ______________ |  |

**Observational checklist for assessing breastfeeding technique.**

| **S/no.** | **Variable** | **Criteria for variables** | **Yes** | **No** |
| --- | --- | --- | --- | --- |
| 1. | Positioning | 1. Baby's body close to the mothers |  |  |
|  |  | 1. Baby's head and body straight |  |  |
|  |  | 1. Baby's mouth and body are facing the breast |  |  |
|  |  | 1. The whole body supported by mother |  |  |
| 2. | Attachment | 1. More areola is seen above the baby’s top lip |  |  |
|  |  | 1. Baby’s mouth has a wide open |  |  |
|  |  | 1. Lips are flanged out/turned out ward |  |  |
|  |  | 1. Chin is pushed into the breast and the nose is clear of the breast |  |  |
| 3. | Suckling | 1. Slow sucks |  |  |
|  |  | 1. Deep sucks |  |  |
|  |  | 1. Sometimes pausing |  |  |

## Amharic Version Questionnaire

ቃለ መጠይቁ የተካሄደበት ቀን (ቀን/ወር/ዓመት):_______________________________________

የጤና ድርጅቱ ስም: ____________________ የመጠይቅ መለያ ቁጥር:_____________________

የቃለ መጠይቅ አቅራቢዉ ስምና ፊርማ: ስም:___________________ ፊርማ፡___________

የተቆጣጣሪዉ ስምና ፊርማ: ስም፡___________________ ፊርማ:____________

**ክፍል አንድማህበራዊ ና ስነ ህዝብ ጉዳይ መረጃ**

| መ/ቁ | ጥያቄ | መልስ ና መለያ ቁጥር | | እለፍ | |
| --- | --- | --- | --- | --- | --- |
| 101 | እድሜሽ ስንት ነዉ (በዓመት) | _________________ | |  | |
| 102 | ብሔርሽ ምድን ነው | 1. አሪ 2. አማራ 3. ወለይታ 4. ሌላ | |  | |
| 103 | ሀይማኖትሽ ምድን ነው | 1. ፕሮቴስታንት 2. ኦርቶዶክስ 3. ሙስሊም 4. ካቶልክ 5. ሌላ | |  | |
| 104 | የጋብቻ ሁነታ | 1. ያገባ 2. ያላገባ 3. የተፋታ 4. የሞተባት 5. ሌላ | |  | |
| 105 | ስራሽ ምንድነዉ | 1. የቤት እመቤት 2. የመንግስት ሠራተኛ 3. መግስታዋ ያልሆነ 4. ነጋዴ 5. የቀን ሠራተኛ 6. ሌላ | |  | |
| 106 | የት/ት ደረጃ | 1. ያልተማረ 2. መጻፍና ና ማንበብ የሚችል 3. መጀመርያ ደረጃ የጨረሰ 4. 2ኛደረጃየጨረሰ 5. 2ኛናመሰናዶት/የጨረሰ 6. ኮሌጅ /ዩኒቨርሲቲ | |  | |
| 107 | የመኖርያ ቦታ | 1. ገጠር 2. ከተማ | |  | |
| 108 | የቤተሰብ ብዛት | _________________ | |  | |
| **ክፍል ሁለት ዉልድና የህፃናት ጤና ሁኔታ / ጉዳይ/** | | | | | |
| 201 | እርግዝና ብዛት | | _______________ | |  |
| 202 | በህይወት የተወለዱ ህጻናት ብዛት | | ______________ | |  |
| 203 | ሞቶ የተወለደ ህፃን ነበረሽ | | 1. አዎ 2. የለም | |  |
| 204 | ጨቅላ ህፃን ሞቶብሽ ያዉቃል | | 1. አዎ 2. የለም | |  |
| 205 | እርግዝና ክትትል ነበረሽ ወይ | | 1. አዎ 2. የለም | |  |
| 206 | ወደ ጤና ድርጅት ለቅድመ ወሊድ ክትትል ስንት ጊዜ ሄደሻል? | | _________________ | |  |
| 207 | እርግዝናዉ የታቀደ ና የተደገፈ ነዉ ወይ | | 1. አዎ 2. አይደለም | |  |
| 208 | ስለ ጡት ማጥባት ሙያ በ እርግዝና ወቅት ትምህርት ወስደዋል? | | 1. አዎ 2. አይደለም | |  |
| 209 | የመጨረሻውን ልጅ የወለድሽበት ቦታ የት ነዉ | | 1. ሆስፒታል 2. ጤና ጣቢያ 3. ቤት | |  |
| 210 | የመጨረሻዉ ልጅ የወለድሽበት መንገድ | | 1. በማህፀን በር 2. በ ቀዶ ጥገና 3. በመሳሪያ ታግዤ | |  |
| 211 | ጤና ተቋም ከወለድሽ ድረ ወልድ ላይ ስለ ጡት ማጥባት ሙያ ትምህርት አግኝተሻል ወይ | | 1. አዎ 2. የለም | |  |
| 212 | ከ6 ወር በፊት ከጡት ዉጭ ምግብ ትሰጭያለሽ ወይ | | 1. አዎ 2. አልሰጥም | |  |
| 113 | ለልጁ ተጨማር ምግብ ጀምረሻል ወይ | | 1. አዎ 2. አልጀመርኩም | |  |
| 214 | የጡጦ ምግብ ታጠብያለሽ ወይ | | 1. አዎ 2. አይደለም | |  |
| 215 | ጡትሽ ላይ የጤና ችግር አለ ወይ | | 1. አለ 2. የለም | |  |
| 216 | አዎ ከሆነ | | 1የጡትጫፍ መሰጠቅ  2የጡት መቁሰል  3 የጡት ማበጥ  4 የጡት ጫፍ ወደ ውስጥ መግባት  5 ሌላ | |  |
| 217 | የመጨረሻዉ የእርግዝና እርዝማኔ | | 1. በቀኑ የተወለደ 2. የተራዘመ የእርግዝና ግዜ 3. ከግዜዉ በፊት የተወለደ | |  |
| 218 | የህፃን ጾታ | | 1. ወንድ 2. ሴት | |  |
| 219 | የህፃን ክብደት | | _________________ | |  |
| 220 | የህፃን ዕድሜ | | _________________ | |  |
| 221 | ህፃን ስወለድ የነበረዉ ክብደት | | _________________ | |  |

## Arigna version questionnaire

ጎይሲን ጀመርሸቃክ

ጤና ድርጅትንት ናሚ_______________________ጎይሲንት ፋይዳ_________________________

ጎይሲዳቢት ናሚ_____________________ ፊርማ፡_______________________

ሀዲ ሸድን ሽዳብት ናሚ___________________ፊርማ፡_______________

የዲውላቅ ኧታ ዶቅቲኬ ሀቅንቲ መረጃ

| **መ/ቁ** | **ጐይሲ** | **ማሲከ ፓሺ ፋይዳ** | **ቃሊካ** |
| --- | --- | --- | --- |
| 101 | ቦናንት ሜሜ | ----------- |  |
| 102 | በህሪ አንተ ሀር | 1. አሪ  2. አማራ  3. ወለይታ  4. ሌላ |  |
| 103 | ሄማኖት አንተ ሀር | 1. ፕሮቴስታንት  2. ኦርቶዶክስ  3. ሙስሊም  4. ካቶልክ  5. ሌላ |  |
| 104 | ታሚ ጎክ ሀስኒ | 1 ተይታ  2 ታያቃብ  3 ቡልታ  4 ደይት  5 አብ |  |
| 105 | ፒክሻን አንተ ሀር | 1 አረ ማ  2 መንግስት አሪ ፒከሻ  3 መንግስት ተማትኪደ  4 negada  5 kena ken pikshidab  6 abe |  |
| 106 | ትምርት ደረጃ | 1 ተማርካቢ  2 ናበበኪ ጻፍከ እስዳብ  3 መጀመሪ ማኪሻብ  4 ቃስተምሲ ደረጃ ማክሽካብ  5 ቃስተምሲ መሰናዶ ማክሽካብ  6 ኮሌጅ ማክሽካብ |  |
| 107 | ሀቢ ዶቅዴ | 1 ከተማ  2 ገጸር |  |
|  | ቤተሰብ ሜሜ |  |  |
| የዲ ካስተምሲ አቲመተከ የኒስታ | | | |
| 201 | ሲርማ ሜሜ | _________________ |  |
| 202 | ናፍሲክ አተርሳክድ ነሲ ሜሜ | _________________ |  |
| 203 | ደሲ አተርሳክድ ሜሜ | 1 ይየ  2 ሃይየ |  |
| 204 | አኪ አትረሲምሽ ደቶ | 1 ይየ  2 ሃይየ |  |
| 205 | ሲርመነት ኪትትል ታ | 1 ይየ  2 ሃይየ |  |
| 206 | ብረን ሲረማት ክትትል መምግዛ ሲ |  |  |
| 207 | ሲረመና አቀደረ ተነደትከ | 1 ይየ  2 ሃይየ |  |
| 208 | ሲረማ ሃዳኪነከ አሚ ጋሲ ጎኪተማረታ | 1 ይየ  2 ሃይየ |  |
| 209 | ማቅነታ የኒሲነም ሃማ አጸ | 1 ሆስፒታል  2 ጤና ጣቢያ  3 ኤሪየ |  |
| 210 | ማቅነታ የኒሲነም ሃረካ አጽ | 1 ማይጸንት ፉለተክ  2 ኖረቲ ሃይሲ ዣጊክ  3 መሳረክ ክሊሽይሶ |  |
| 211 | ጤና ጣቢያ አጽዮ ቡሪ ክትትል ዘን አሚ ጋሲ ጎክ ትምረት ተይታዮ | 1 ይየ  2 ሃይየ |  |
| 212 | አረፊ ላት ቢሪዘን ሬይ አብ እሲሚ እመታ | 1 ይየ  2 ሃይየ |  |
| 213 | አበንዳ እሲሚ እሚ የታዮ | 1 ይየ  2 ሃይየ |  |
| 214 | ቱቶ እሲሚ አመታዮ | 1 ይየ  2 ሃይየ |  |
| 215 | አሚሃንተዛ ርየ አብ ችግር ዶኮ | 1 ይየ  2 ሃይየ |  |
| 216 | ይየ ሃጋይሲ | 1 አሚት ዱሪዘን ሃየ  2 አሚት ዘና ሙዳ ዲዲ  3 አሚት ዘና አቲሚ  4 አሚት ዱሪ ዋር ማቲ አርድ  5 አብ |  |
| 217 | ማኪንታ ሲረምንት | 1 ሰጽንክ አቲርሳቃብከ  2 ዶክሲ አቲርሳቃብከ  3 ቢረዛን አቲርሳቃብከ |  |
| 218 | ዪነሲነት ጾታ | 1 አንጀ  2 ማ |  |
| 219 | ዪነሲነት ድጽሚ | _________________ |  |
| 220 | ዪነሲነት ቦን | _________________ |  |
| 221 | ዪነሲነት | _________________ |  |
